# An Augmented Modulated Deep Learning Based Intelligent Predictive Model for Brain Tumor Detection Using GAN Ensemble

**DOI:** 10.3390/s23156930

**Published:** 2023-08-03

**Authors:** Saswati Sahoo, Sushruta Mishra, Baidyanath Panda, Akash Kumar Bhoi, Paolo Barsocchi

**Affiliations:** 1 School of Computer Engineering, KIIT Deemed to be University, Bhubaneswar 751024, India; sushruta.mishrafcs@kiit.ac.in; 2 LTIMindtree, 1 American Row, 3rd Floor, Hartford, CT 06103, USA; baidyanathpanda@gmail.com; 3 Directorate of Research, Sikkim Manipal University, Gangtok 737102, India; akashkrbhoi@gmail.com; 4 KIET Group of Institutions, Delhi-NCR, Ghaziabad 201206, India; 5 Institute of Information Science and Technologies, National Research Council, 56124 Pisa, Italy

**Keywords:** brain tumor, deep learning, generative adversarial network, machine learning, PGGAN, soft voting

## Abstract

Brain tumor detection in the initial stage is becoming an intricate task for clinicians worldwide. The diagnosis of brain tumor patients is rigorous in the later stages, which is a serious concern. Although there are related pragmatic clinical tools and multiple models based on machine learning (ML) for the effective diagnosis of patients, these models still provide less accuracy and take immense time for patient screening during the diagnosis process. Hence, there is still a need to develop a more precise model for more accurate screening of patients to detect brain tumors in the beginning stages and aid clinicians in diagnosis, making the brain tumor assessment more reliable. In this research, a performance analysis of the impact of different generative adversarial networks (GAN) on the early detection of brain tumors is presented. Based on it, a novel hybrid enhanced predictive convolution neural network (CNN) model using a hybrid GAN ensemble is proposed. Brain tumor image data is augmented using a GAN ensemble, which is fed for classification using a hybrid modulated CNN technique. The outcome is generated through a soft voting approach where the final prediction is based on the GAN, which computes the highest value for different performance metrics. This analysis demonstrated that evaluation with a progressive-growing generative adversarial network (PGGAN) architecture produced the best result. In the analysis, PGGAN outperformed others, computing the accuracy, precision, recall, F1-score, and negative predictive value (NPV) to be 98.85, 98.45%, 97.2%, 98.11%, and 98.09%, respectively. Additionally, a very low latency of 3.4 s is determined with PGGAN. The PGGAN model enhanced the overall performance of the identification of brain cell tissues in real time. Therefore, it may be inferred to suggest that brain tumor detection in patients using PGGAN augmentation with the proposed modulated CNN technique generates the optimum performance using the soft voting approach.

## 1. Introduction

**Figure 1:**
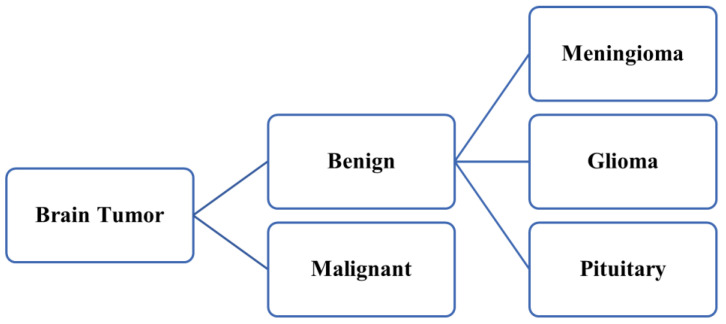
Categorization of brain tumor.

Accurate diagnosis of brain tumors in the initial phase is very essential to averting the global death rate due to cancer-related illness [1]. Proper segmentation of brain tumors is indeed a key challenge and time-consuming procedure in the modern era due to the constantly increasing number of brain tumor patients all across the world [2]. To diagnose brain tumors accurately, radiologists adopt magnetic resonance (MR) imaging methods to visualize the inner shape of a patient’s brain securely and reduce the chances of any surgery [3,4]. MR imaging helps radiologists provide necessary data regarding a patient soft cell tissues that assist the doctors while handling cases related to the brain tumor in the real-time diagnosis of a patient [5,6,7]. Accurate segments of MR images are required to help doctors with the correct identification of brain tumors using multifarious computer-aided medical instruments. While the acquired MR images are being segmented accurately, the brain tumors may be categorized into two diverse categories, which are malignant (cancerous) and benign (non-cancerous) [8,9]. Benign (non-cancerous) brain tumors are further categorized into three diverse classes, which are meningiomas, gliomas, and pituitary tumors. The precise and timely identification of tissues is one of the major concerns and demands high focus. It is a tedious task because of the varied traits of the segmented brain tissues, such as the dimension and shape of the tissues as well as their accurate position with the intensity of the gray level [10,11,12]. Figure 1 illustrates the categorization of the brain tumor. 

**Figure 2:**
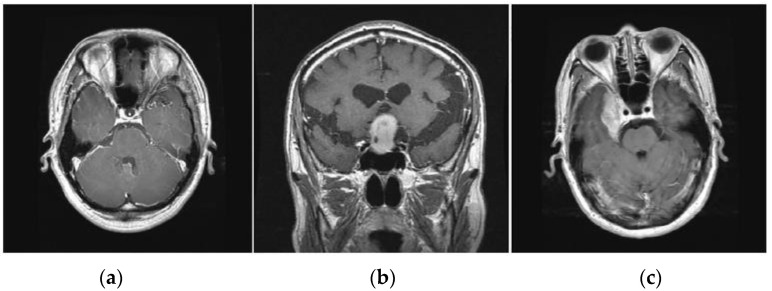
Various types of brain tumors (**a**) Glioma (**b**) Pituitary (**c**) Meningioma.

Several brain tumor detection techniques aid in detecting brain tumors at early stages. These techniques may help clinicians continue a pragmatic diagnosis of patients as per the requirements. However, the majority of them have accuracy and latency limitations [13,14]. In addition, the data size used in the study is sparse and suffers from inadequate training [15]. The accurate detection and categorization of normal tissue as well as abnormal tissue is a major challenge. Brain tumor detection in the initial phase is essential for the effective diagnosis of the patients immediately. The main motivation to conduct this research was to overcome the major limitations of the existing work. Encouraged by the success of deep learning, various deep neural networks have proven to help overcome the limitations in the classification and detection of brain tumors. However, regardless of encouraging results, the scarcity of large volumes of data, generating realistic images completely different from the original ones, and the quality of synthesized images remain challenging issues to be addressed [16]. So, there is a need for a computationally interconnected advanced framework that can provide more reliability and accuracy with less response time delay so that it can be diagnosed with more precision. The initial diagnosis mainly emphasizes monitoring the symptoms of the patient facing any disorder related to brain cell tissues [17] Figure 2.

Gliomas are positioned within the cerebral hemispheres; however, they may also appear in certain parts of the human brain. Pituitary tumors are positioned within the sella turcica, a minor depression within the skull that covers the pituitary gland. Meningiomas are positioned within the meninges, the membranes that cover the brain as well as the spinal cord.

Furthermore, such approaches can assist clinicians in the identification of brain cell tumors quickly and efficiently. This study aims to demonstrate both the strengths and major limitations of previously suggested brain tumor identification and categorization techniques as addressed in previous works. It also provides a comprehensive assessment of the examined literature analysis and reveals fresh investigation perspectives. Furthermore, this article proposes a new augmented modulated deep learning-based intelligent predictive model for brain tumor detection using a GAN ensemble.

### Research Gap and Motivation

Existing clinical tools and ML-based models developed for brain tumor screening are limited in their potential to precisely identify varied-dimension tumors, particularly in the growing phase. This is due to the brain tumors diversity, variation in tumor development, and complex brain structure. Therefore, there must be developed an improvised model to detect brain tumors accurately. 

The construction of an advanced and more robust predictive model for brain tumor identification utilizing the GAN ensemble approach has huge potential to address the limitations of existing approaches and improve the diagnosis process. GANs are a type of artificial intelligence (AI) technique that can be utilized to generate realistic images. By modulating the GANs along with ensemble learning, it is feasible to build a predictive model that is more precise and informative than conventional brain tumor screening approaches.

The construction of the proposed model would have a remarkable impact in the area of brain tumor diagnosis research. It would assist the clinical experts and allow accurate detection of brain tumors in the beginning stages, which may lead to enhanced treatment results. In addition to this, this proposed predictive model may be utilized to build novel diagnostic instruments and enhance the understanding of brain tumor development.

This research study has the potential to make a valuable contribution to the area of brain tumor research. The construction of this predictive model for brain tumor detection using a GAN ensemble would allow for early detection of tumors, which would lead to enhanced treatment results. In addition to this, the model may be utilized to build novel clinical instruments and enhance the understanding of brain tumor development.

The main contributions of the research study are as follows:

- In this research analysis, an integrated performance evaluation of different popular GAN approaches is done in the context of brain tumor symptom detection.
- This study proposes a novel augmented modulated deep learning-based advanced predictive model utilizing the voting-based GAN ensemble for the early detection of brain tumors.
- Enhanced hybrid CNN model with PGGAN architecture produced the best outcome recording, i.e., an optimum accuracy, precision, recall, F1-Score, and NPV of 98.85, 98.45%, 97.2%, 98.11%, and 98.09%, respectively. Additionally, the least time latency of 3.4 s is noted with the proposed hybrid model.
- The outcome of the implementation analysis through various performance parameters demonstrates that PGGAN is the most suitable augmented method for data generation with sufficient diversity in comparison with other GANs.

## 2. Literature Review and Background Study

Multifarious investigations have been conducted during the last decade for the early diagnosis of brain tumors, along with proper categorizations. However, the existing methods have various limitations due to several limitations, such as lower precision and accuracy in numerous cases. 

### 2.1. Relevant Research in Context to Machine Learning (ML) for Brain Tumor Analysis

**Figure 3:**
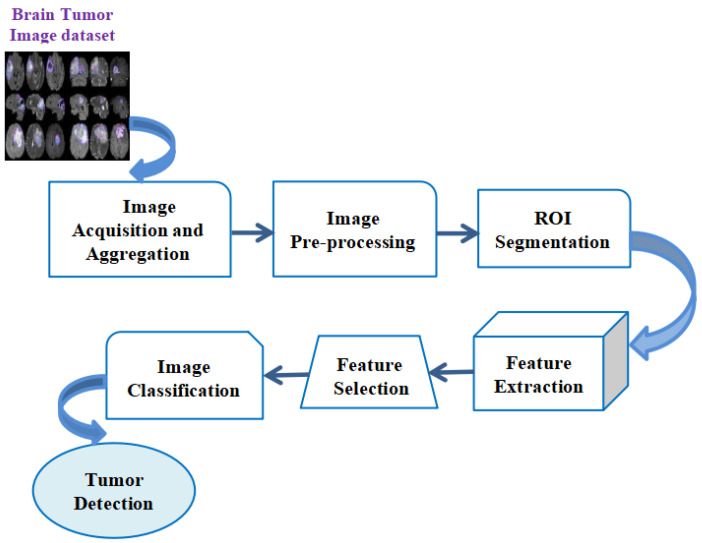
Sample depiction of brain tumor recognition utilizing conventional Machine Learning.

This section describes the existing research carried out in the field of early detection of diverse types of brain tumors by utilizing machine learning (ML) protocols in the real-time diagnosis of patients. Figure 3 depicts the sample depiction of brain tumor recognition utilizing conventional ML. In conventional ML, the first step is the pre-processing phase, which includes the utilization of the filter for removing unwanted noise for the picture contrast improvement and effective and easy identification of brain tumors. 

In the initial pre-processing phase of the input pictures, the picture is first divided into multiple segments by utilizing segmentation methods such as the cluster-rooted scheme, thresholding, and edge-rooted split procedure. Later, the segmentation procedure is performed, and the multiple features extraction procedure is conducted to be rooted on the colored datasets, texture-based contrasts, and dimensions from a region of convergence (ROI). After that, multifarious principal features are to be evaluated by utilizing the feature-chosen method, namely the PCA (Principal Component Analysis) or the statistical examination. The selection of features is a procedure that aids in minimizing the input of a variable within the model by utilizing multiple relevant datasets and helps to eliminate the noise from the datasets. This is a very important procedure for the automated selection of some relevant features for the ML model based on these kinds of issues. Then, the chosen features are to be utilized to feed into the ML classifier, namely the neural network (NN) or the support vector machine (SVM). The chosen classifier may utilize the capture features vector with the destination class labels for determining the optimum boundary that separates every class. While the chosen ML-rooted classifier is to be trained, this could be utilized for the classification of novel, unknown datasets for the evaluation of the classes. The ML classifier would take the optimal features vector as a feed, and the outcome would be the object classes. Table 1 illustrates the methods utilized based on ML protocols for the detection of brain tumors.

### 2.2. Relevant Research in Context to Deep Learning for Brain Tumor Analysis

Proper brain tumor classification might, in turn, lead to the most suitable medical therapy and, as a result, increase human longevity significantly. Numerous existing approaches have been used to conduct a substantial study on accurate brain tumor tissue identification. However, current approaches for identifying brain tumors have several limitations, such as the lower accuracy of methods used in real-time recognition of MR images to identify and categorize brain tumors appropriately for early treatment of patients. The deep learning models are realistic and more pragmatic in terms of performance measures of various parameters, namely the sensitivity, specificity, or precision in real-time as compared to ML methods due to multifarious reasons, such as minimizing the subjective assessment for brain tumor identifications. Therefore, deep learning models are more secure and capable of training large datasets in real time. 

**Figure 4:**
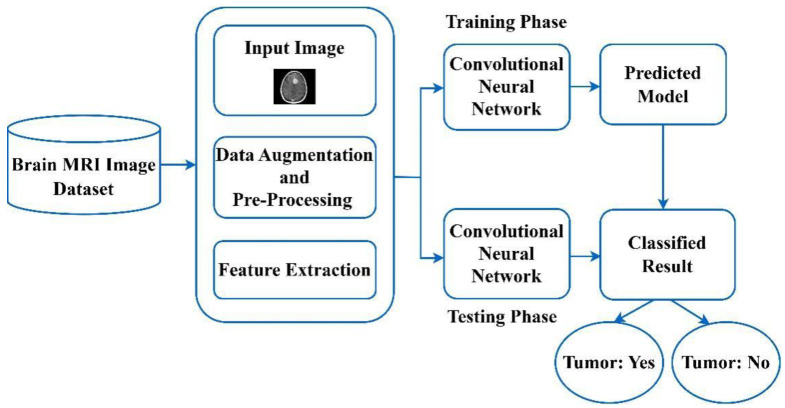
A functional block diagram of brain tumor categorization utilizing the CNN method.

CNN has been widely utilized, and the famous deep learning (DL) rooted prototypical utilized globally to segment and perform categorization procedures of the medicinal pictures. Figure 4 depicts the functional block diagram of brain tumor categorization utilizing the CNN technique. The CNN-rooted brain tumor categorization process is segregated into two diverse stages. One stage is known as the training stage, and another one is recognized as the testing stage. Each brain tumor picture is to be segregated into diverse classes by utilizing the names of the tags, like the tumor as well as without the tumor brain picture, etc. During the entire training stage, various kinds of processes are conducted, such as augmentation, picture categorization, picture accurate pre-processing, along with picture feature extraction to make the prediction prototypical. During the pre-processing stage, picture rescaling is to be employed to alter the picture dimension. Lastly, the CNN technique is to be utilized for automated categorization of the diverse kinds of brain tumors. Table 2 depicts the popular deep learning-based approaches used earlier for the recognition of diverse brain tumors. 

**Figure 5:**
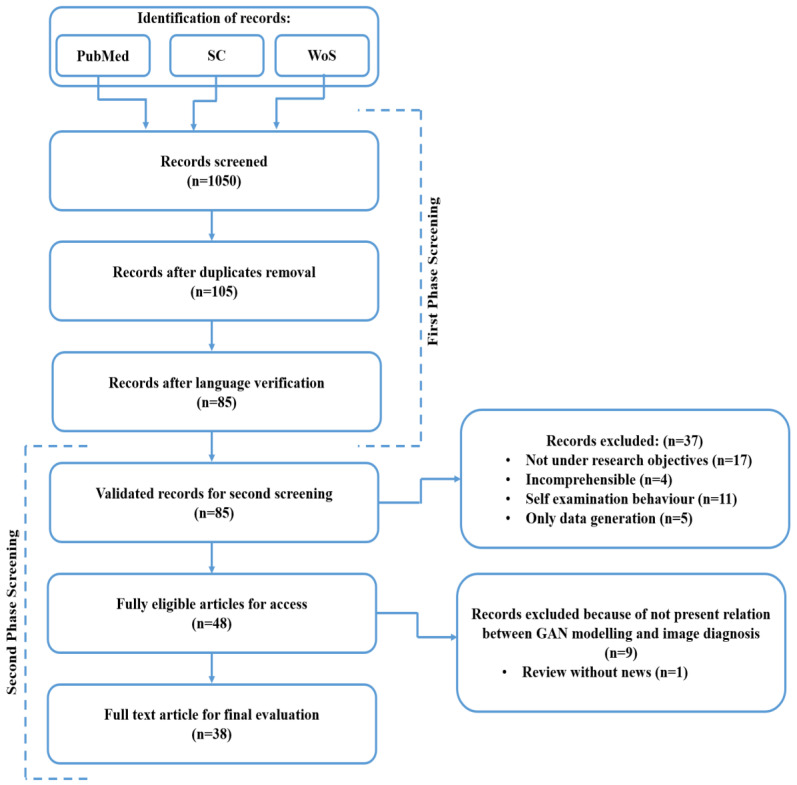
A systematic procedure for relevant publications analysis.

This section provides a detailed procedure for our search strategy. In this study, the authors conducted an organized literature examination that aligned with the existing papers. For finding the pragmatically relevant work done by various authors, we performed our search utilizing diverse databases, which include PubMed, Scopus (SC), and WoS (Web of Science). The primary aim to perform the search properly was to recognize the most pragmatic studies among the GAN applications and diverse other recent functionality. We have searched the databases by utilizing the diverse kinds of specific synonyms of the major keywords, which are primarily related to the GAN. Further, we utilized various kinds of Boolean operatives, including the “GAN classification” OR “Types of GANs” OR “GAN categorization” OR “GAN for brain tumor” OR ‘GAN in the medical field’ AND ‘GAN for brain tissue recognition’. Furthermore, the WoS and Scopus databases have been fully utilized for identifying the multiple existing published papers by numerous authors in this field during the last decade all across the world. Figure 5 depicts the systematic procedure for relevant publication analysis.

In this research, a detailed analysis has been conducted utilizing the eligible research and review articles for an organized systematic review as well as a meta-analysis through PRISMA methodology. The PRISMA approach includes a set of algorithms for rational concepts and multiple longitudinal studies, which involve formatting and writing specifications. In this literature review, there is a three-step method that involves research question formation, guidelines to accept or discard the research, and a review of articles and online databases. 

**Figure 6:**
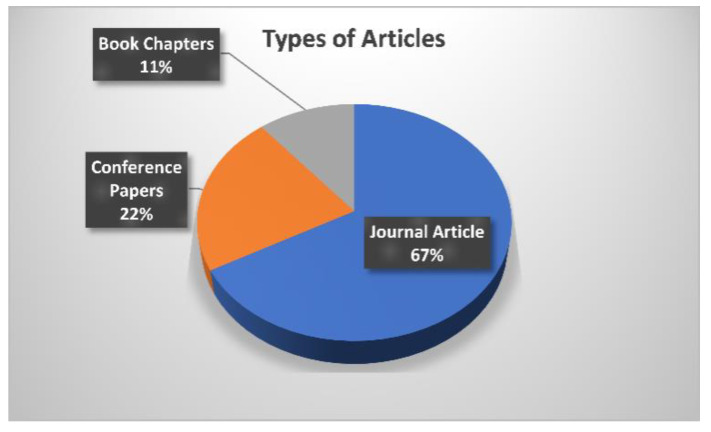
Analysis of articles used in the review process.

Figure 6 illustrates the analysis of articles used in the review process. This review involves 67% journal articles, 22% conference papers, and 11% book chapters in the initial screening and selection of the papers. The inclusion criteria are set on certain questionnaires, e.g., articles aimed at resolving issues with brain tumor identification utilizing AI and ML, search queries should involve the title of the scientific study, the abstract, and the entire body of reputed peer-reviewed journals or conferences. The exclusion criteria involve research articles published with identical findings, studies that are not relevant to brain tumor detection or segmentation, as well as scientific studies published in other languages rather than English. 

In this study, GAN-based literature searching results offered 1050 diverse records, which are classified through the varied published articles that are associated with the main aim of this analysis. Initially, the recognition of the diverse records was done by utilizing the selected three databases, namely PubMed, SC (Scopus) as well as WoS. After, a total of 1050 screened records were obtained by using the databases. For accurate searching over the databases, we divided the entire screening procedure into two separate screenings, namely first-phase screening and second-phase screening. The records after the duplicate’s removal were identified (n = 105). The records after the language verification have been measured (n = 85). In the second phase screening procedure, validated records for the second screening were (n = 85), fully eligible articles for access were (n = 48) and a full-text article for the final evaluation was found (n = 38). Furthermore, the author filtered out the articles for review by considering the three classes of articles, such as original manuscripts, review manuscripts, and analytical manuscripts. 

As observed from the existing literature survey, there exist research models and diverse approaches by different investigators in recent years for significant brain tumor categorization and identification that could be followed through diverse phases, namely prediction of the outcome, categorization, and diagnosis plan. However, such conventional developed models and screening approaches for brain tumor identification have certain restrictions in terms of accuracy in image categorization, increased execution time, and high computational overhead. Therefore, there is a need for the development of a new advanced deep learning-based intelligent predictive model for brain tumor detection, which can detect brain tumor symptoms with enhanced accuracy and minimal time delay, saving the time of clinicians in the real-time screening of the patients and perform diagnosis of patients in a more significant manner. 

## 3. Working Principle of GAN

The tumor has been regarded as the most common cause of death across the globe. The broad diversity in sickness intensity, length of sickness, tumor position, numerous levels of susceptibility, or chemotherapeutic medicines might have been to blame for low tumor categorization. During the last few years, the proportion of individuals suffering from brain cell tissues has increased dramatically. Proper brain tumor categorization may lead to the best appropriate medicinal care and therefore considerably improve human longevity. The GAN (generative adversarial network) is a very powerful and correct approach that is capable of synthesizing novel MR images through the aid of latent vectors in real time. GAN is an ML-rooted framework that comprises a plurality of models such as generators and discriminators. 

**Figure 7:**
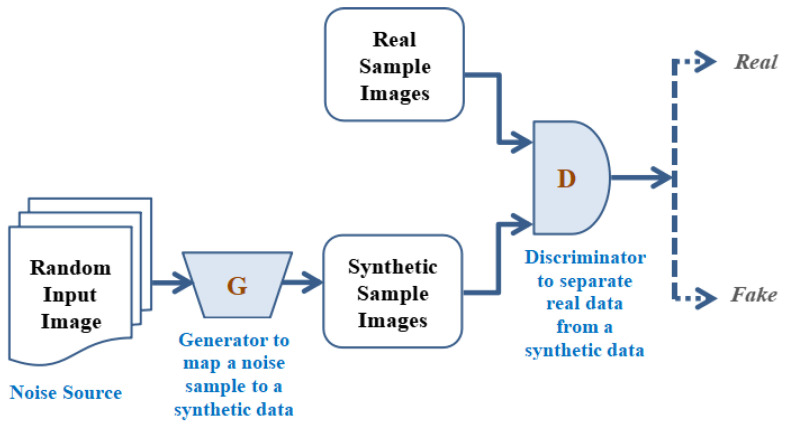
Working principle of Generative adversarial Network.

The GAN works primarily on the diverse three regulations; the beginner one is to make generative prototypical learning and datasets, which may be produced engaging numerous probabilistic illustrations. In the secondary stage, the model training process may be completed in any kind of inconsistent scenario. In the third phase, utilizing DLNN (Deep Learning rooted Neural Network) and utilizing artificial intelligence (AI) rooted protocol to train the entire system. Figure 7 depicts the overall working process of GAN. In the working of the GAN, a selected random input image is translated to the generator to map a noise sample to synthetic data and the synthetic sample images are to be generated. These synthetic sample images are to be used to apply to the discriminator where the obtained real sample images and synthetic sample images are compared and the discriminator separates the real data from the synthetic data in an accurate manner. Furthermore, based on the effective comparison this discriminator classifies the images into real and fake categories. Table 3 illustrates the types of GANs that form the GAN ensemble in our research. The GAN model architecture refers to the specified design of the model, which involves, layers types, the total number of layers used as well as the connection among different layers. The model training process means, the steps used in model training such as datasets utilized in model training, selection of the hyperparameters as well as specific stopping criteria utilized to stop the model training process, accordingly. 

The following is a systematic explanation of the GAN functioning.

Step 1: The operators have to be made utilizing the generator as a means of discriminative network commencing accurate datasets distribution in real-time. 

Step 2: Now training the system such that the overall networks accountability level could be enlarged as well as entire discriminator networks may be misled employing creating such applicants, which are not fully combined for instance, which are a stagnant fragment of the distribution of the entire dataset.

Step 3: After that, the entire data could turn into personalizing training datasets for the entire discriminator in real time. 

Step 4: To train the entire sampling data, which is accessible until the required accurateness is to be attained. 

Step 5: To train the generator for producing the applicants while the entire discriminator is to be misled soon as this is fed with the arbitrary feed in it to perform the required processes in real-time. 

Step 6: At last, back-propagation is to be applied for the entire generators along with the discriminator where in previous produces improved pictures, as well as later one, is to be accomplished at declining synthetic pictures. 

## 4. Materials and Methods

This research analysis deals with the development of a novel hybrid and computationally augmented deep learning model for brain tumor detection in patients using GAN. This section discusses the dataset used in the study and the proposed framework for brain tumor assessment. 

### 4.1. Dataset Used in the Study

In this work, the authors utilized available public data suggested by Cheng [60]. This dataset comprises overall 3064 CE-MR images, which include the three diverse kinds of brain tumors (for instance, the meningioma and glioma along with the pituitary) from 233 diversely ill candidates. The entire images utilized in the presented data were chosen in 2-Dimensional and not a single image was chosen in 3-Dimensional volume images in real-time performance measurement of the suggested model. In this investigation, authors encompassed all three of the accessible planes (for instance, the axial as well as coronal, along with the sagittal) images from the presented data. To balance the dataset, there has been selected oversampling technique. The oversampling technique duplicates minority class samples for matching multifarious majority class samples. It is achieved through duplicating samples, arbitrarily or by employing a method, namely the synthetic minority oversampling technique (SMOTE). Table 4 depicts the partition of the brain tumor dataset into training, validation, and test samples. 

### 4.2. System Configuration

In this work, for experimentation work authors utilized the Ubuntu OS (Operating System) configuration: 18.04.2 LTS, which is maintained through the GeForce Nvidia Giga Texel Shader eXtreme (GTX) 1080 Graphics processing unit (GPU). We have written the entire codes using the PyCharm tool in Python, version v3.8.0 including numerous external libraries, for instance, Keras and TensorFlow, Matplotlib, and many more. 

### 4.3. Proposed Methodology 

**Figure 8:**
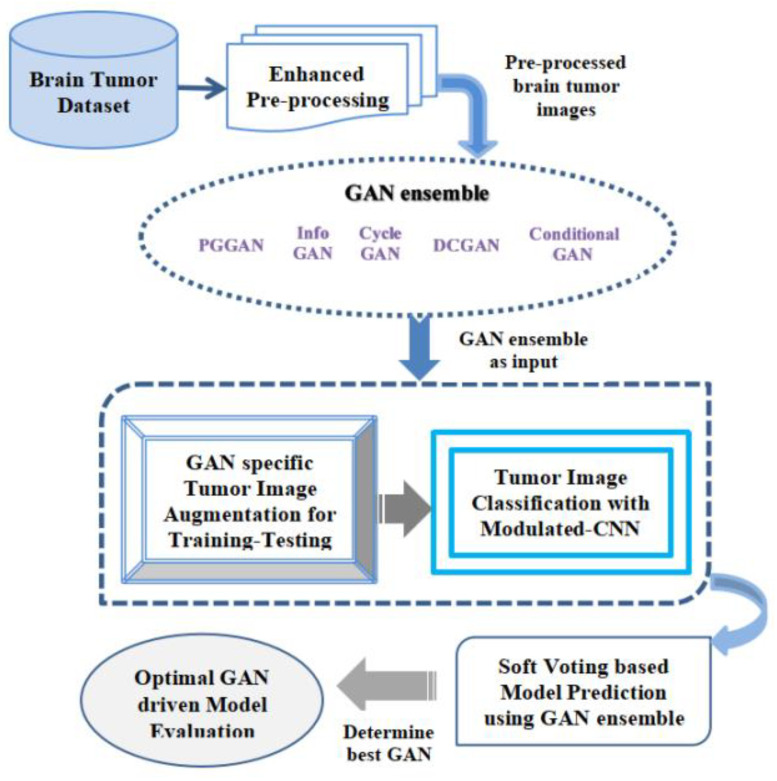
Proposed GAN ensemble Hybrid CNN-based Predictive Model.

Multifarious classes of GANs have diverse outcomes according to real-time implementations. The few commonly recognized classes of GAN are DCGAN (Deep Convolutional-Generative Adversarial Network), Conditional GAN, Cycle GAN, Info-GAN, and PG (progressive growing) GAN [48]. In this research work, the authors recognized PGGANs as one of the suitable approaches for the feature extractions of the brain tumor from multiple images as its accuracy, F1-score, and all other similar parameters are better in comparison to the other GANs. Figure 8 illustrates the proposed GAN ensemble Modulated CNN-based predictive model. The GAN ensemble used herein refers to a combined group of diverse GANs, which include PGGAN, Info GAN, Cycle GAN, DCGAN, and Conditional GAN. The key reason for the selection of the GAN ensemble approach in our study is to provide more stability to the model and to find a suitable GAN out of diverse selected GAN classes to detect and classify the brain tumor more accurately and effectively in real-time patients’ diagnosis and aids to the clinicians during the patients screening. The main reason to select the GAN ensemble in comparison to the single GAN is that the GAN ensemble is capable of pragmatic modeling the distribution of the normal dataset, thereby aiding in the detection of brain tumor anomalies in a significant manner.

The explanation of the working procedure of the novel hybrid model is described in this section. The initial phase of the suggested model obtains the real-time MR image datasets of the various individuals for brain tumor detection effectively. Brain tumors are constantly becoming a serious concern around the world, as they are a group of irregular cells. These abnormal brain cell tissues are continuously becoming dangerous and cause deaths. For proper identification of the kind of brain tumors, namely the glioma, pituitary as well as meningioma, an accurate dataset sample was collected for accurate performance evaluation using the suggested GAN ensemble hybrid CNN-based predictive model. After obtaining the datasets, the enhanced pre-processing of the collected datasets samples is to be done efficiently with a high degree of precision. In this enhanced pre-processing phase, the entire acquired raw datasets are effectively transformed into clean datasets. The enhanced pre-processing procedure used here mainly consists of four separate phases, which include datasets quality assessment, cleansing of the obtained datasets, the transformation of the dataset, and minimization of the datasets. This image dataset pre-processing stage was applied for making the obtained datasets sample stable and highly reliable for suggested ensemble prediction analysis in real time. In this entire stage, numerous datasets pre-processing methods along with the accurate selection of diverse features of the pictures are to be done in a required manner constantly. The datasets pre-processing describes the translation of accessible datasets before this being fed in the suggested GAN ensemble Hybrid CNN-based predictive model. The authors primarily utilized methods, namely control missed values and obtained datasets’ accurate encoding. In the end, we opted dataset normalization technique to scale up the features in a required manner. After the pre-processing phase, the entire image datasets are to be translated towards the GAN ensemble in real time. Herein, multiple GANs are employed such as the PGGAN, Info GAN, Cycle GAN, DCGAN, and conditional GAN. The entire accessible image datasets were then processed through the chosen GAN ensemble for accurate performance evaluation. In this stage, the entire processed output from the GAN ensemble is further mapped for GAN-specific tumor image augmentation for performing the training and testing of entire accessible datasets for the proper brain cell tissue recognition in real time. This is a critical step, and a high degree of precision is required during the entire training and testing phase of the datasets. The datasets augmentation procedure in the proposed GAN ensemble Hybrid CNN-based predictive model helps in rising the datasets amount artificially as means of producing fresh dataset points through existing datasets. After the completion of the brain tumor picture augmentation that is training and testing, then the collected datasets are translated further for the brain cell tissues categorization procedure employing the modulated-CNN-based approach embedded within the suggested model, which provides more accurate outcomes in the image’s classification procedure. Once the categorization procedure is completed utilizing the suggested modulated-CNN-based approach, the received datasets are further input for the soft voting approach, which is based upon the suggested model prediction utilizing the GAN ensemble approach. In the soft computing approach used in the proposed model, the best GAN is chosen based upon various specified performance parameters such as accuracy, precision, as well as F1-Score, and Sensitivity, accurately with a high degree of precision for the optimal GAN-driven model evaluation in real-time. It is observed that PGGAN is computed to be the most promising GAN for fast, reliable, and accurate image classification during brain tumor categorization. 

#### 4.3.1. Pre-Processing Algorithm for the Skull Stripping

The obtained brain tumor dataset is translated to the image pre-processing segment. The pre-processing of the obtained dataset is to be done by utilizing the skull stripping along with the contrast enhancement. Table 5 illustrates the abbreviation used in the pre-processing algorithm for the Skull stripping.

The automated skull stripping of the MRI pictures has been recognized very important segment in MRI pictures’ efficient analysis. The accurateness of the human skull stripping protocol distresses multifarious apps, namely the tumor segmentation, pre-surgical arrangement, etc. This is a simple procedure for the removal of non-cerebral nerves, namely the skull and eyeball from MRI brain pictures. The pre-processing algorithm for the skull striping is illustrated below. In this algorithm, the MRI brain picture (I_MR_) denotes the input and the skull-stripped picture (I_MRstripped_) denotes the outcome. The detailed algorithm procedure is illustrated as follows. Initially, MR input images were binarized utilizing Otsu’s technique. This approach attains the threshold level which reduces in class-variation amongst two distinct categories. With the help of obtained binary images, the biggest connected component (CC) was received. The brain portion is the biggest CC within the obtained image. This biggest CC is further dilated by utilizing the 3 × 3 squared structured components to maintain the minimal brain data within the output images. Furthermore, the resultant image pixel was superimposed along with the accessed input picture to attain the stripped skull image, effectively. 

Step 1: I_MR_ ← Input MRI picture of a brain

Step 2: I_MR bin_ ← Otsu (I_MR_)

Step 3: Conv ← Highest associated element (I_MR bin_)

Step 4: Conv’ ← Conv ⨁ SE_t1_

Repeat

Step 5: Conv’_Kt_ ← Conv’_Kt−1_ ⨁ SE_t2_ $\cap \;I_{{MRI}^{c}}$

Until

Conv’ _Kt_ = Conv’ _Kt−1_

I_MRIstripped_ ← Superimpose (Conv’ _Kt_)

#### 4.3.2. Pre-Processing Algorithm for the MRI Picture Contrast Improvement

During the pre-processing stage of the MRI pictures, it is to be found that at certain times the contrast of the pictures is below a threshold limit. Table 6 illustrates the abbreviation used in the pre-processing algorithm for the MRI picture contrast improvement.

For an effective visualization of the MRI pictures, the hardware, and software-level picture improvement, must be evaluated. The improvement of the MRI pictures contrast is a procedure for enhancing the visual elements of the received picture such that this could be appropriate for multifarious applications. The contrast improvement procedure provides additional clarity to the MRI pictures, which offers a solution for additional analysis and makes overall analysis much easier as well as fast in comparison to the other kinds of existing schemes. In this algorithm, the skull-stripped picture (I_MRIstripped_) is considered as an input and the contrast-improved picture (I_MRIimproved_) is the outcome. The key advantage of this specific pre-processing algorithm for the MRI picture contrast improvement rather than traditional methods is that it is faster, more accurate, and easy to implement and consumes very less time in comparison to the conventional approach. The main steps of this pre-processing algorithm are described as follows. In the beginning phase, the algorithm attained the complement of the applied stripped skull MRI image. Furthermore, the closing function has been applied over the complement picture as means of disk structured component. Later in the next phase, the complement is computed of the last outcome. Further, the mathematical function attains the variance among the resultant as well as the real picture. Lastly, the real picture has been added along with differentiated pictures for obtaining the contrast-improved images. 

Step 1: I_MRIstripped_ ← Skull stripping picture

Step 2: $I_{{MRI}^{c}}\leftarrow$ (L_t−1_)− I_MRI_

Step 3: C_t_← $I_{{MRI}^{c}}\times$ SE_t2_

Step 4: D_t_ ← (L_t−1_) − C_t_

Step 5: I_MRIdiff_ ← I_MRI_ − D_t_

Step 6: I_MRIimproved_ ← I_MRIstripped +_ I_MRIdiff_

#### 4.3.3. Classification Using Modulated-CNN

**Figure 9:**
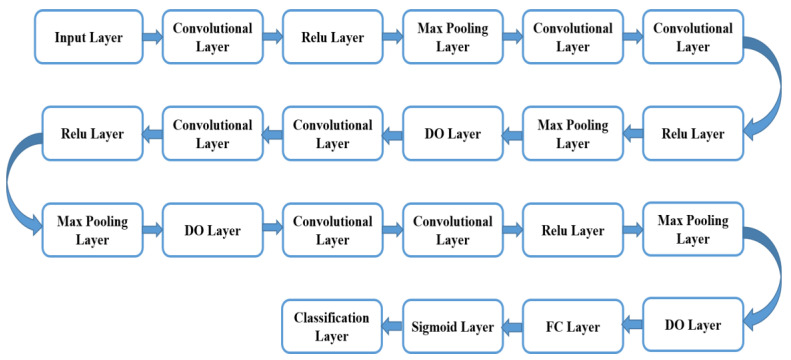
Illustrates the block diagram of the suggested modulated CNN classifier.

Figure 9 illustrates the block diagram of the suggested modulated CNN classifier. For the modulated CNN classifier, the utilized algorithm is given below. In this enhanced algorithm, we opted for T_image,_ labeled as an input, and the predicted outcome of the modulated CNN classifier is described through the output. Table 7 illustrates the abbreviation used in classification using Modulated-CNN. The entire steps of the suggested modulated CNN classification are illustrated as follows: In this classification process, the testing datasets are fed along with the Nk number datasets to the training network. Further, the predicted score is recorded for the testing process. The dataset Pt1 predicts through the model of modulated CNN. Later, the training begins using the classified data to compute the model outcome in terms of Malignant or Benign classification, effectively. 

Step 1: To Feed testing datasets along with the N_k_ number datasets to the training network

Step 2: To obtain the predicted scores

Step 3: For every testing, dataset Pt1 ← predicts via the model of modulated CNN

Step 4: Var X left ← 0 as well as Var X right ← 0

Step 5: if Pt1_is_ found “Malignant” then

Step 6: Pt1 _right_ ← Pt1 _right_ +1

Step 7: Pt1 _left_ ← Pt1 _left_ +1

Step 8: if Pt1 _right_ > Pt1 _left_

Step 9: Classification ← “Malignant”

Step 10: Else 

Step 11: Classification ← “Benign”

Step 12: halt

In this work, the modulated CNN classifier has been built rooted on VGG-19, which includes seven convolutional layers; max-pooling layers were selected at four, as well as a full linked layer at one. The convolutional layer is indeed one of the key building elements of the suggested modulated CNN classifier. The modulated CNN approach is effective and beneficial to attain enhanced accuracy outcomes in comparison to conventional techniques. 

In this research, the CNN classifier used is customized to attain more interpretability and accuracy. This classifier is selected and customized based on the complexity of the task as well as the available dataset size. In addition to this, various hyperparameters, namely learning rate, epoch, and batch size, were adjusted for optimal tuning in model training and testing. 

This convolutional layer comprises multifarious filter parameters that require learning via appropriate training. The chosen filter dimensions are generally taken as small in comparison to the real picture. Each chosen filter convolves along with the picture and generates the map of activation. The max-pooling is a very significant operation that chooses the highest components via the feature map region enclosed through the filter. The fully linked layer aids in the compilation of the datasets extracted via the last layer to produce the end outcome. Furthermore, there have been used 4 ReLU (Rectified Linear Unit) layers in this suggested modulated CNN. The key benefit of the ReLU function utilization over the multiple activation functions is that there is not any requirement to activate the entire employed neuron at the same moment. The sigmoid layer has been utilized after the fully linked layer in place of softmax for the two-fold categorization. The sigmoid layer is employed as the second last layer the suggested modulated CNN classifier as it aids in classifier outcome alteration in the probability score. After every layer, a dropout (DO) layer has been installed for minimization of the overfitting [50]. This DO layer aids to ignore the arbitrarily chosen neuron during the training procedure. Through the simplification of the VGG-19 setup, the hyper-parameter turning procedure is more controllable. The VGG-19 network infrastructure is the CNN, which is to be trained on large-picture datasets through the ImageNet database. The VGG-19 network comprises 19 layers and may categorize the pictures into thousand diverse object classes, namely the keyboard, eraser, sharper, and many more. Consequently, this network infrastructure has learned opulent features illustration for multifarious image datasets. The total nineteen layers of the VGG-19 include three fully linked layers as well as 16 convolutional layers, which aid in the effective categorization of the pictures in diverse object groups. This approach is a suitable for picture categorization owing to the utilization of multifarious 3 × 3 filters inside every convolutional layer. 

**Figure 10:**
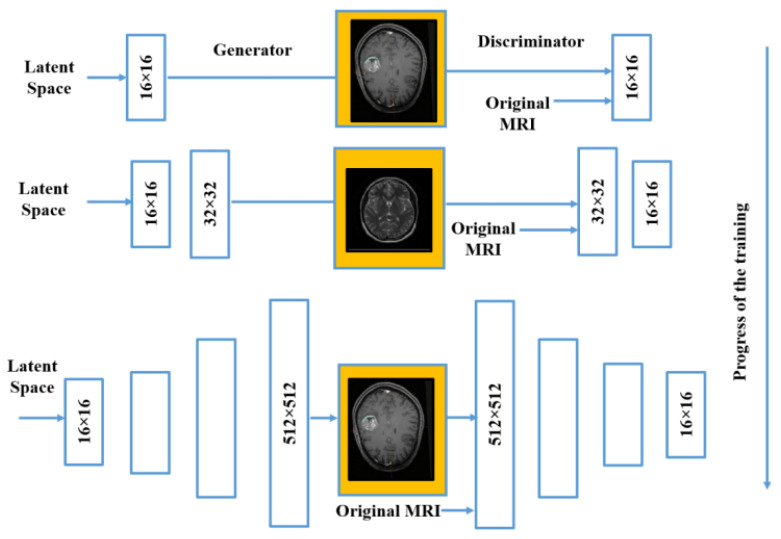
PGGAN structure in real-time training for brain cell tissue generator.

To enable clinicians to choose the appropriate kind of diagnosis as well as care strategy, properly forecast destiny, and effectively establish follow-up strategies, precision in categorizing tumor types is necessary. This investigation work has been carried out by utilizing the PGGAN architecture, which is rooted in the deep learning prototypical. This verification model comprises three diverse principal phases. The initial phase is known as the data pre-processing phase. The second phase is rooted in the deep learning prototypical to augment all the features of the feed in the picture accurately. The third phase of the illustrated model is the MR input image categorization phase. In the third phase, the VGG19 features extractor has been implemented for accurate and proper identification of all the features of the MR input images in real time. Figure 10 illustrates the structure of the PGGAN for 512 × 512-pixel MR picture in real-time training for the brain cell tissue generator. 

## 5. Implementation Results and Discussion

Brain tumor cases are constantly increasing around the world. However, there are numerous clinical methods for determining the brain cell tissues in the diagnosis with the help of MR images taken by the radiologist. However, most of the existing methods have diverse limitations during early detection of the brain cell tissues, which is a huge problem and demands more attention towards novel research for correct recognition and segmentation of the brain tumor pragmatically. The authors of this paper present a hybrid and enhanced brain tumor assessment model based on the GAN ensemble and demonstrate PGGAN as the most promising approach for extracting characteristics of a brain tumor from real-time pictures. 

Different evaluation indicators are utilized for validating the efficacy of the suggested methodology [49,50]. From the confusion matrix, four parameters are derived. True positives (TP) denote accurate predictions of the desired target affected by a brain tumor. True negatives (TN) represent accurate predictions of individuals not affected by a brain tumor. False positives (FP) highlight the inaccurate predictions of a normal individual shown as a brain tumor patient. False negatives (FN) depict inaccurate predictions of the desired target as normal individuals.

The accuracy rate denotes the proportion between accurate values under prediction and the cumulative values predicted, which is shown in Equation (1) as follows:

(1) $$\mathrm{Accuracy}=\frac{\mathrm{TP}+\mathrm{TN}}{\mathrm{TP}+\mathrm{TN}+\mathrm{FP}+\mathrm{FN}}$$

Precision represents the overall ratio of accurately predicted values to the total accurate values that includes both true and false predicted outcomes, which are denoted by Equation (2) as follows:

(2) $$\mathrm{Precision}=\frac{\mathrm{TP}}{\mathrm{TP}+\mathrm{FP}}$$

Recall illustrates the entire proportion of accurate likelihoods to the summation of accurate positively predicted values and inaccurate negatively predict outcomes, which are shown in Equation (3) as follows:

(3) $$\mathrm{Recall}=\frac{\mathrm{TP}}{\mathrm{TP}+\mathrm{FN}}$$

F-Score represents the weighted mean of the values generated from the computation of precision and recall metrics, which is shown in Equation (4) as follows:

(4) $$F-\mathrm{Score}=\frac{\mathrm{TP}}{\mathrm{TP}+1/2(\mathrm{FP}+\mathrm{FN})}$$

**Figure 11:**
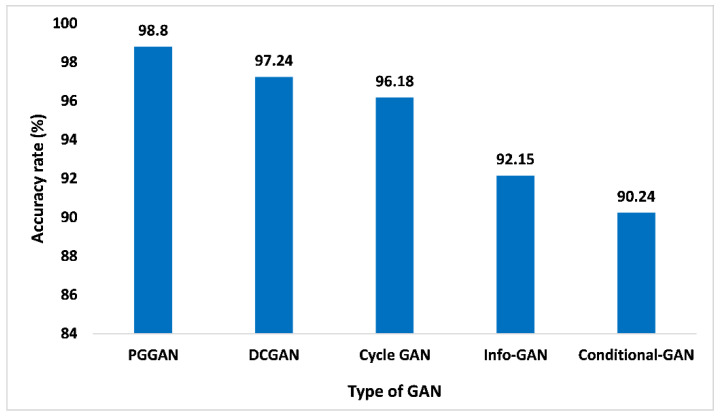
Accuracy analysis for different types of GAN.

The accuracy analysis of different kinds of GAN models is depicted in Figure 11. PGGAN achieved a 98.8% accuracy rate, which is higher than the other competing GANs. DCGAN with an accuracy of 97.24%, Cycle-GAN with an accuracy of 96.18%, info-GAN with an accuracy of 92.15%, and Conditional GAN with an accuracy of 90.24%. The reason for such high accuracy lies in the fact that PGGAN is very stable where the model size increases incrementally. The training and validation of the suggested GAN ensemble intelligent predictive model for brain tumor identification and categorization are done with higher precision to measure the model accuracy level on distinct GANs. The outcomes are recorded in real-time on the chosen datasets for model training and validation. The accuracy was measured on distinct images to identify the glioma, meningioma, and pituitary categories of the tumor. We selected three planes of the images, i.e., sagittal, coronal, and axial. For glioma, meningioma, and the pituitary, the total number of images were 1426, 708, and 930, respectively. The accuracy of the proposed model is measured very accurately as we segregated the entire chosen image datasets into three classes, i.e., training data, validation data, and testing data. The training data was considered at 60%, as well as validation data at 20% and the model testing data at 20%. The key advantage of the proposed GAN ensemble model is that it is capable of identifying and classifying the glioma, meningioma, and pituitary tumor from chosen images considering distinct planes in real-time, which is a significant advantage of the proposed GAN ensemble model over the existing models, which provides a lower accuracy level considering all the planes of the images.

**Figure 12:**
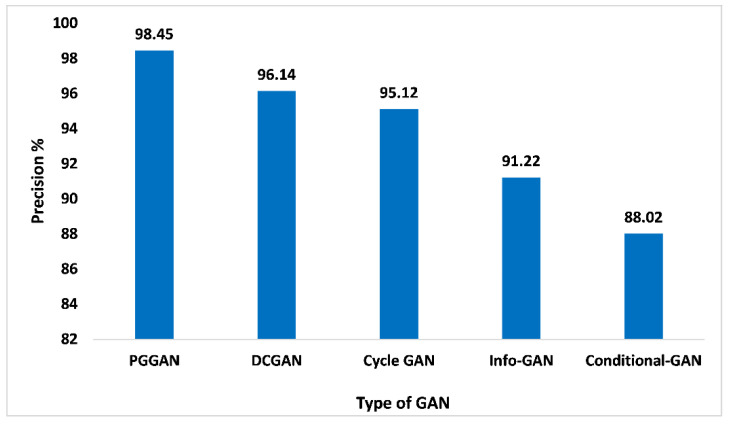
Precision analysis for different types of GAN.

The precision matrices are a very important indicator for determining the proposed GAN ensemble model performance. The precision is referred to as overall true positive numbers divided by overall positive predictions, which means true positive numbers and false positive numbers added. When analyzing Figure 12, with 98.45% of precision, PGGAN attained the maximum, which is high compared to other GANs. While Precision values for DCGAN, Cycle-GAN, Info-GAN, and Conditional-GAN are 96.14%, 95.12%, 91.22%, and 88.02%, respectively. The incremental nature of PGGAN enhances the performance of the model. Thereby, the proposed GAN ensemble model offers a pragmatic and higher precision value on the PGGAN when compared with the other GAN approaches.

**Figure 13:**
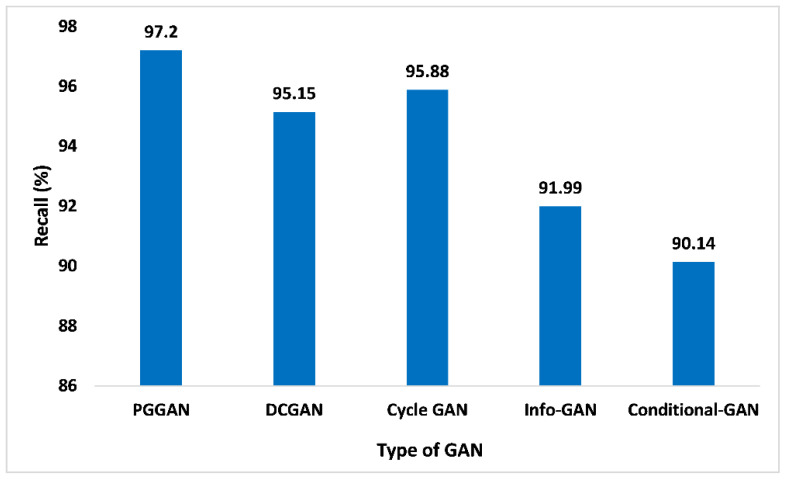
Recall analysis for different types of GAN.

The recall performance matrices are not just restricted to binary categorization issues. Within the imbalanced categorization issue with the above two categories, the recall is evaluated as the addition of true positives across the overall categories divided by the addition of true positives and false negatives across every category. In addition to this, recall is indeed a very important metric that aids in quantifying the accurate positive numbers forecasted out of the entire forecast of positive. Similar to the precision matrices, which may comment over accurate forecasting of positives out of each forecasting of positives, the recall matrices may offer a sign of failed forecasting of positive. Figure 13 presents the outcome of PGGAN based on recall value is 97.2%, which is the maximum in comparison to DCGAN, Cycle-GAN, Info-GAN, and Conditional-GAN, which are 95.15%, 95.88%, 91.99%, and 90.14%, respectively. Thus, the proposed GAN ensemble model offers pragmatic advantages and a higher recall value when compared with other GANs. 

**Figure 14:**
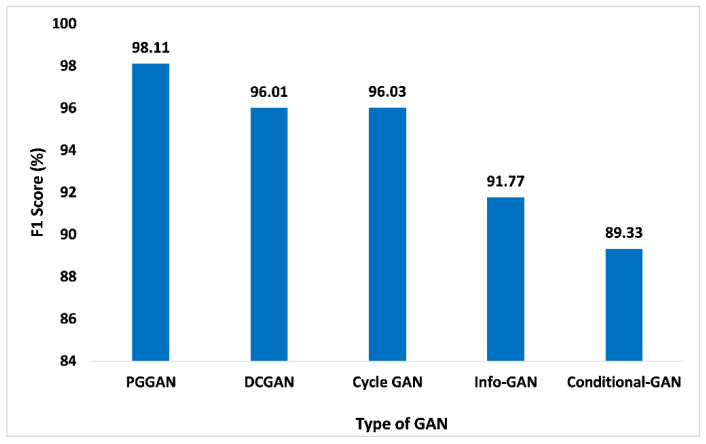
F1-Score analysis for different types of GAN.

The F1-score is another significant performance matrix that combines the recall and precision of the classifier within one metric by considering the harmonic mean. This performance metric aids in comparing the performance of the proposed GAN ensemble model with distinct GANs. Similarly, performance analysis based on the F1-score is given in Figure 14. Compared to other GANs, PGGAN attained the maximum 98.11% F1 score. DCGAN, Cycle GAN, Info-GAN, and conditional-GAN attained the F1-score 96.01%, 96.03%, 91.77%, and 89.33%, respectively. Thus, the measured F1-score value of the proposed GAN ensemble model for PGGAN is improved and pragmatic when compared with the other GANs. 

**Figure 15:**
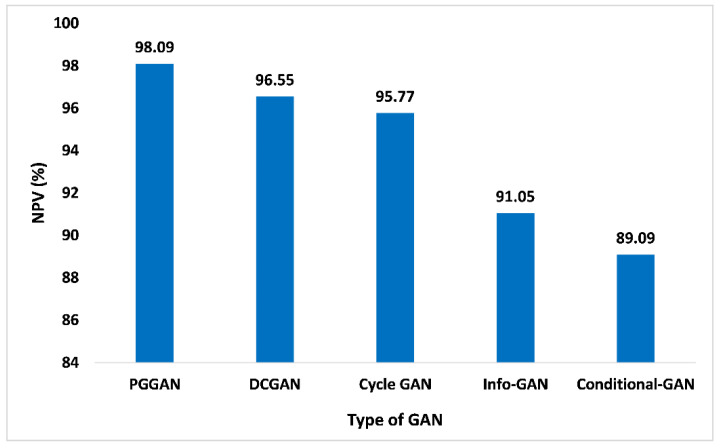
NPV analysis for different types of GAN.

The NPV is described as the proportion of forecasted negatives that are true negatives. In addition to this, it demonstrates the likelihood that the forecasted negative is indeed the real negative. Figure 15 shows the NPV parameter of PGGAN is 98.09%, which is excellent among other competitive GANs. The NPV parameter values for DCGAN, Cycle-GAN, Info-GAN, and Conditional-GAN are 96.55%, 95.77%, 91.05%, and 89.09%, respectively. Thus, the proposed GAN ensemble model offers a higher NPV value over the PGGAN in comparison to the distinct other GANs such as the DCGAN and others. 

**Figure 16:**
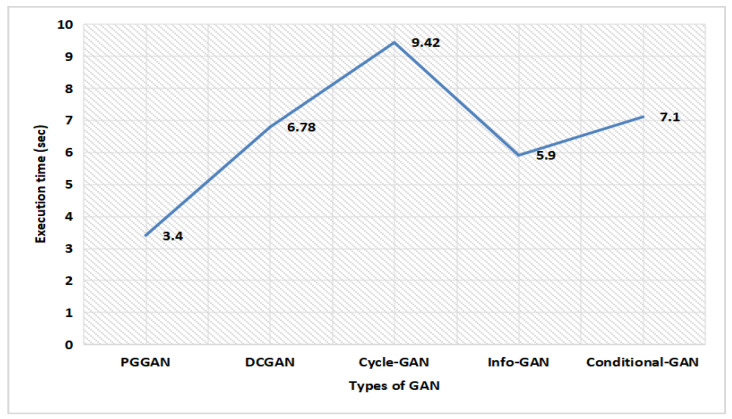
Execution time analysis for different types of GAN.

Figure 16 illustrates the execution time analysis for different types of GAN. The progressive growth of the generator and discriminator causes a lower execution time delay for PGGAN. The proposed GAN ensemble model consumes very minimal time on PGGAN, which is only 3.4 s, while the DCGAN, Cycle-GAN, Info-GAN, and Conditional-GAN consumes more time, which is 6.78 s, 9.42 s, 5.9 s, and 7.1 s, respectively, which is far greater in comparison to the PGGAN. Thereby, the proposed GAN ensemble model predicts that on PGGAN, the proposed model consumes very minimal time. 

**Figure 17:**
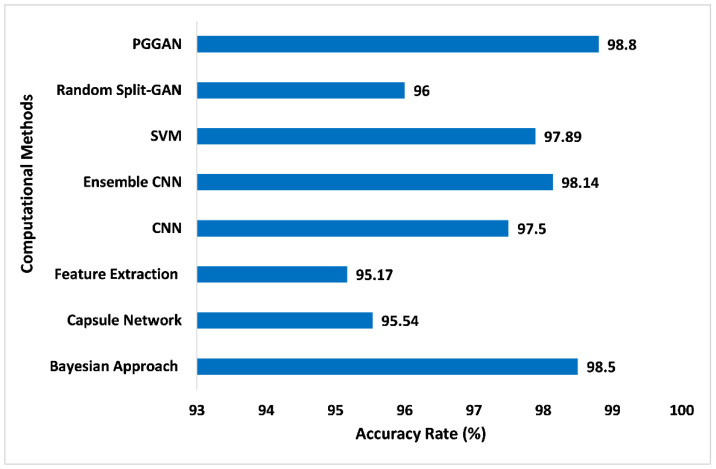
Comparison of accuracy for different computational methods with PGGAN [61,62,63,64,65,66,67].

Figure 17 illustrates the comparison of accuracy for different computational methods with PGGAN. The PGGAN has been globally recognized for the addition of the GAN training procedure, which can assist to train the multifarious generator prototype and may produce huge higher-quality pictures. It entails beginning with an extremely tiny picture as well as gradually accumulating stages to raise generator prototypical output dimensions and discriminator prototypical input dimensions until the required picture dimension is reached. Due to the diverse appearance and complexity of tumors, PGGAN provides satisfaction and high accuracy in the sense of tumor detection and will provide pre-treatment to the patients, so they can be cured. The proposed GAN ensemble model accuracy has been measured and compared with the other computational methods. In the analysis of proposed model accuracy with PGGAN and other methods, it has been found that the accuracy on the Bayesian approach [61], Capsule network [62], Feature extraction [63], CNN [64], Ensemble CNN [65], SVM [66], and Random Split-GAN [67] were 98.50%, 95.54%, 95.17%, 97.50%, 98.14%, 97.89%, and 96.0%, respectively. However, the proposed GAN ensemble model offers an accuracy level of 98.80% with the PGGAN, which is higher than the other computational methods. 

Table 8 presents the performance of the proposed model through various performance metrics such as accuracy, precision, recall, F1-score, and NPV with different portioning of training, testing, and validation ratio of the MRI brain tumor dataset. The best performance was observed for the ratio of 60:20:20 with scores of 98.8%, 98.45%, 97.2%, 98.11%, and, 98.09% for accuracy, precision, recall, F1-Score, and NPV, respectively.

Table 9 illustrates the performance metric evaluation of the proposed model for different diseases. The proposed model performance is computed for different disease classes and assessed through performance metrics such as accuracy, NPV, precision, recall, and F1-Score. The best optimal performance was observed for brain tumors with scores of 98.8%, 98.09%, 98.45%, 97.2%, and 98.11% for accuracy, NPV, precision, recall, and F1-Score, respectively. 

Table 10 presents a cumulative analysis of performance metrics for different types of GANs. As observed from the table, the optimum outcome is generated using PGGAN. Here, 98.8%, 98.45%, 97.2%, 98.11%, and 98.09% are the recorded values for accuracy, precision, recall, F1-score, and NPV, respectively.

In this experimental study design and implementation, a few of the identified insights into the limits and possible error sources of the proposed model for brain tumor detection have been identified. A few of the key limits and possible errors can involve the limited size of the datasets, challenges to managing the overfitting, etc. Although this proposed GAN ensemble model for brain tumor detection attains very optimized results, there can be limits to the research work and possible sources of the errors, which demand attention to resolve them. In addition to this, the generalizability of the proposed GAN ensemble model should be computed on larger datasets and in diverse clinical settings based on diverse tumor classes and sizes to determine its applicability beyond the input dataset it has been trained on.

## 6. Conclusions and Future Scope

Brain tumor has become one of the most prevalent causes of mortality all around the world. This research work designs a new hybrid augmentation-based modulated CNN architecture using a soft voting approach. GAN ensemble augmented brain MRI image data samples and the augmented dataset are fed into a modulated CNN model for the classification of images. PGGAN was found to be the most promising GAN technique, generating the most optimal values in performance metrics using a soft voting approach. The accuracy, precision, recall, F1-Score, and NPV were computed to be 98.8%, 98.45%, 97.2%, 98.11%, and 98.09%, respectively. It also recorded a very low execution time delay of only 3.4 s with PGGAN. The time required by the DCGAN, Cycle-GAN, Info-GAN, and Conditional-GAN is 6.78 s, 9.42 s, 5.9 s, and 7.1 s, respectively, which is higher in comparison to the PGGAN approach. As a result, a deep classification model using PGGAN with modulated CNN can be very effective in assisting medical professionals to assess brain tumor symptoms in patients. 

## Figures and Tables

**Table 1:** Relevant Conventional ML methods for the detection of brain tumors.

Methodology Utilized	Novelty	Benefits	Limitations
In this article, the authors conducted an experiment for performance computation of numerous ML algorithms for effective categorization of brain tumors [18].	It is found that multilayer perceptron along with logistic regression provides the optimized outcomes for segmentation and classification tasks.	Improved diagnosis, enhanced planning of patient’s treatment, as well as usages of multimodal information.	Selection of the ideal features for recognition of the diverse classes seems to be a more complex as well as time taking procedure due to the lack of generalization possibilities.
In this article, the authors adapted the Extended Kalman Filter (EKF) as well as SVM to determine the brain tissues automatically [19].	This novel model ensemble an Extended Kalman Filter (EKF) as well as Support Vector Machine (SVM) to categorize brain tumors in MR images.	Better classification performance for positive brain tumor images, automatic diagnosis as well as effective segmentation.	Possibilities of misclassification in the categorization procedure. Additionally, the present approach is computationally more complex owing to the usage of conventional region-growing algorithms.
In this work, the authors proposed an improved brain tumor classification approach using a template-based K-means (TBK) algorithm [20].	Initially, the important features were extracted utilizing the super pixels as well as principal component analysis (PCA) that aids to determine brain tumors effectively.	Possible implementation applicability in the area of medical image processing for the detection and diagnosis of brain tumors.	This suggested approach is capable to enhance the margins concerning multifarious features earlier chosen. However, this approach also provides lower classification accuracy.
In this work, the authors adopted a fuzzy classifier and SVM-based machine-learning methodology for MR image segmentation [21].	In this proposed model, centroid optimizations, namely Social Spider Optimization (SSO) as well as Grey Wolf Optimization (GWO) integrated along with the Genetic Algorithm (GA) for improving the accuracy level.	An improved analysis of the potential impact of multiple features as well as parameters on the performance of ML-based systems for the determination of brain tumors.	This framework is less robust to alter different settings, namely slice thickness, imaging parameters, slice, contrast, etc.
In this work, authors proposed a framework for MR image classification using the Logistic Regression (LR) method [22].	This suggested framework functions pragmatically on enhanced LR algorithm for varied dimensions of tumor classification.	An optimized outcome is measured on limited data and a more generalized system in terms of computing cost.	This scheme is incapable to function along with multifarious dimensions clusters and varied densities.
In this work, authors utilized, Logistic Regression (LR) rooted ML protocol for MRI picture categorization for early brain tumor identification [23].	In this work, logistic regression is used as the ML algorithm to classify MR images in different classes based on the absence or presence of brain tumors.	Possible applicability in the area of medical imaging for early identification as well as diagnosis of brain tumors.	In this work, while the alteration is done in the acquired data, another novel preparation data is needed.
In this paper, the authors used two protocols for the classification of brain cell tissues such as Glioma tumors along with the accurate prediction of individual survival [24].	The novelty of the proposed research article lies in the integration of SVMs for the segmentation of brain tumors in MR imaging and the prediction of survival of patients rooted in segmentation outcomes.	Prediction of effective survival of patients rooted in the segmentation outcomes, which may aid in the personalized diagnosis of brain tumors.	It is to be found that accuracy is low with this approach for classifying brain tumors in very early stages.
In this paper, the authors used logistic regression as well as Gaussian Naïve Bayes for determining the initial site for the brain cells tissues [25].	The novelty of the proposed research is that it involves an ML model that utilizes MR-based radiomic features to forecast the primary site of brain metastases. This is a pragmatic improvement over existing methods, which have relied on clinical features, alone.	This proposed framework can forecast the primary site of brain metastases and has an accuracy of 80%, which is significantly improvised than existing methods.	It is to be found that huge search issues for discovering the closest neighbor as well as datasets storage.
In this article, the authors used a novel categorization strategy that includes a random forest approach along with the delta radio mic feature in real-time [26].	The novel feature of the proposed research is that it integrates an ML model which employed radiomic features derived from dynamic susceptibility contrast-enhanced (DSC) MR imaging for classifying glioblastoma into high-grade as well as low-grade tumors.	This proposed model was found capable to categorize glioblastoma tumors with an accuracy of 94%, which is significantly enhanced in comparison to existing approaches.	This work is incapable of early tissue detection due to the large time requirements of the period as well as memory.
In this work, the author developed a different technique for brain tumor detection that is rooted in the SVM (Support Vector Machine) and applied with the help of SVM kernels [27].	This research proposes a framework to improve the performance of SVM to categorize MR brain images in benign as well as malignant tumors.	The enhanced pre-processing methods were found able to enhance the contrast of the image, which enables the SVM classifier in tumor boundaries identification, effectively.	The proposed approach seems to be asymmetric concerning the quality of earlier tissue indication in brain cells.
In this work, the author used MR (Magnetic Resonance) Images to classify the tumor class based on an automatic segmentation strategy [28].	In this work, an extreme learning machine (ELM) is utilized to classify MR images in diverse categories rooted in the absence or presence of brain tumors.	A more effective as well as accurate system to identify brain tumors in comparison to previous research.	Here, the evaluation of the main element reduces the dimension of the text features.
This research work proposed new brain tumor identification and segmentation scheme centered on the random-forest (RF) classifier [29].	The noises In brain MRI images have been determined as well as minimized in preprocessing phase and then GLCM is extracted using preprocessed MRI images.	Possibility to employ the model in the area of medical imaging to aid clinicians in the early diagnosis of brain tumor patients.	The accuracy of this method seems to be lower as well as a promise of an identical feature vector is impossible.
In this article, the authors proposed another strategy for determining the brain cell tissues according to rooted over the dimensional feature of the MR (Magnetic Resonance) images with the help of ML [30].	In this article, a new method is proposed to assess brain tumors utilizing the shape features of MR imaging through ML. The technique integrates an SVM-based classifier to categorize brain tumors.	The shape features allow us to capture the subtle variance between diverse classes of brain tumors.	Whereas it is found some alteration in the picture data, there is always a necessity for the freshly trained dataset. Therefore, the proposed strategy could only be useful for CT pictures.
In this article, the authors used the decision tree strategy to train multifarious pixels of MR images of the Gliomas class [31].	In this research, a fully automated brain tumor segmentation technique has been proposed that utilizes the ensemble learning method.	This ensemble learning technique obtained an accuracy of 86%, which is found significantly improvised than the accuracy of existing research.	It is to be found that accurate extraction of desired image features is complex due to the higher grouping of the MR images pixels.
In this paper, the authors used diverse strategies combination one is the threshold segmentation and another is the Watershed segmentation protocol for the classification of the brain cells tissues [32].	A new framework is proposed for brain tumor identification, which integrates a combination of threshold segmentation, and watershed segmentation, as well SVM classification.	The present research is a pragmatic contribution to the area of brain tumor diagnosis. It gives a novel as well as more accurate technique to evaluate brain tumors using MR imaging, which may lead to enhanced results.	After analysis of this article, it is to be found that a lower threshold amount seems to prompt the grouping.
In the present article, the author developed a neural network (NN) rooted method for categorization of the brain cells tissues with the help of three diverse phases such as accurate extraction of the features and minimization of the size along categorization pragmatically [33].	Development of a new ML-based framework to classify brain tumors through enhanced neural network architecture.	Possible applicability in medical imaging areas to diagnose brain tumor patients.	In this existing work, multifarious issues have been identified such as numerous pathologies and gathering, also this scheme’s accuracy level is low.
In this article, the authors utilized watershed segmentation through the ML protocol [34].	A brain tumor classification model is proposed, which utilizes a learning-rooted approach. This suggested model utilizes a set of image processing as well as ML-based algorithms to determine brain tumors from MR imaging.	This suggested framework attained an accuracy of 90%, which is significantly enhanced in comparison to the assessed accuracy of other techniques.	This approach is inefficient because the dataset is smaller and only provides the accuracy of a large dataset to determine the cell tissues.
In this work, the authors utilized the GBML (Gradient Boosting Machine Learning) strategy for brain tumor categorization [35].	The proposed technique integrates GLCM, intensity, as well as GLRLM features to illustrate brain MR imaging. The extracted features are then categorized utilizing the GBML to determine the meningioma tumors, presence.	This framework is fully automated which means there is not any requirement of human intervention in screening. This can make it a pragmatic tool for clinical diagnosis.	The major limitation of this work is more prediction time over large images to detect small affected tissues of the brain.
In this paper, the authors developed another method for brain cell tissues through the ASVM (Adaptive Vector Support Vector Machine) [36].	In this work, a combination of intensity, as well as texture features, are used to illustrate brain MR imaging. The obtained features were further categorized utilizing SVMs to identify brain tumors, presence.	The proposed framework allows to identify of diverse brain tumors, involving both benign as well as malignant tumors.	It is to be found that the proposed method has multifarious limitations such as inaccuracy in the detection of the cell tissue along with high computation cost in the training of the large dataset in real-time.
In this article, the authors suggested brain tumor classification by utilizing SVM as well as the K-nearest neighbors (KNN) technique [37].	In this research, a new framework is proposed for brain tumor categorization utilizing the active contours without the edge-rooted segmentation method.	It offers an alternative to conventional edge-rooted segmentation methods.	The major limitation of this suggested approach is the large time consumption in the categorization of the brain tumor effectively.
In this article, the author proposed brain MR image classification using the improved KNN as well as GLCM [38].	In this model, Median Filtering has been adopted for better pre-processing of the MR images.	This suggested approach may be utilized in clinical settings for assisting medical experts in brain tumor patients’ diagnoses.	The major drawback of this suggested KNN-based model is more time consumption in the pre-processing and training phase which are significant limitations of the proposed model.
In this research, a new method for brain tumor identification is developed which is based on the RF classifier [39].	The suggested methodology utilizes the feature optimization approach for the indentation and segmentation of the brain tumor.	This model may be an alternative to conventional segmentation methods.	However, this model also has a few limitations, namely deficient brain tumor segmentation accuracy level and more computational complexity.
In this article, the authors proposed a new framework for brain tumor analysis which is based on Naïve Bayes algorithm [40].	The obtained MR pictures are subjected to pre-processing as well as feature extraction for accurate tumor segmentation.	This suggested framework utilizes the data augmentation approach for increasing sample size as well as minimizing over-fitting possibility.	This model provides evaluation matrix values comparatively lower and implementation of the model in real-time applications seems to be computationally more complex.
In this study, the authors investigated a computer-aided (CAD) system for determining the brain’s tumor [41].	This proposed scheme utilizes the FLAIR modality for determining the abnormality since the irregularity is highly obvious.	This suggested methodology may offer an alternative to a conventional brain tumor as well as glioma-grade categorization approaches.	The key limitation of this model is the huge time consumption in the categorization of images simultaneously to detect the kind of brain tumor effectively.

**Table 2:** Popular Deep Learning based models for recognition of the diverse brain tumors.

Methodology Utilized	Novelty	Benefits	Limitations
In this article, the authors adapted ANN (Artificial Neural Networks) as well as CNN (Convolutional Neural Networks) for monitoring the accurate location of the brain cells tissues along with the performance measurement [42].	In this research, a hybrid ensemble approach based on modified ANN and CNN architecture has been developed for brain tumor classification.	This suggested methodology may be employed in clinical settings to assist the medical practice in brain tumor patients’ diagnosis.	The proposed techniques work fast but consume much time due to the large computational complexity in the prediction of brain cells tissues
In this research paper, the authors proposed another deep-learning protocol for brain cell tissue classifications. In this suggested article brain cells tissues monitoring procedure was done via pre-processing as well as the skull stripping in real-time for evaluation of the brain tissue’s proper segmentation [43].	A modified deep-learning framework has been developed for brain tumor classification and segmentation utilizing MR images.	The proposed framework may provide an alternative to traditional brain tumor segmentation as well as classification techniques.	The major drawback of this work is the lowest performance in MR image segmentation because the performance was measured with the help of F-measure which is incapable to determine the brain cell’s tissues in low densities scenarios.
The authors utilized another deep learning-based strategy for brain cells tissues classification with the help of the FAHS (Fully Automated Heterogeneous Segmentation)-SVM (Support Vector Machine) method [44].	This suggested technique utilizes a modified CNN which is capable to learn the features of brain tumors through MR imaging.	This methodology is simpler in terms of model implementation and deployment process in clinical settings.	This work devises some limitations such as being slow in determining the abnormal organization of the cell’s tissues with the help of MR images.
In this article, the authors adapted CNN as well as the DBN method for brain cell tissue detection [45].	This suggested methodology employs a modified deep learning algorithm to determine the patterns in fused clinical MR images, which involve indicative symptoms of brain tumors.	This suggested framework may offer more insights into the dimensions and location of brain tumors than conventional approaches.	The major drawback of the suggested method is low performance in the case of large dataset processing.
In this paper, the author used the HE (Histogram Equalization) strategy for cells tissues segmentation [46].	In this research, a customized CNN architecture has been developed and integrated with the HE to classify the MR images as malignant or benign.	It is found that this suggested methodology may be more reliable than conventional methods for brain tumor identification.	The major drawback of this work is that it attains less accuracy on the lower contrast MR image.
In this article, the authors utilized Deep-CNN (Deep Convolutional Neural Network) method for brain cell tissues segmentation [47].	This suggested methodology utilizes a customized Deep-CNN architecture as well as an image-processing approach to enhance the accuracy of brain tumor identification.	This suggested methodology may enhance the patient’s lifetime by early identification of brain tumors.	This method is incapable to maintain a huge dimension dataset of MR images.
The author has chosen the CNN (Convolutional Neural Network) as well as ANN (Artificial Neural Network) methods jointly [48].	This suggested framework integrates a depth-wise separable Deep-DCNN to extract and classify the features of brain tumors using MR imaging.	This suggested method may determine a distinct class of brain tumors than conventional methods.	The major restriction of the proposed work is lower efficiency in the case of a complex dataset and consumes more time in the training.
In this article, the authors conducted a review analysis based on DL methods for the categorization of brain tumors [49].	This analysis offers an important insight that may affect the performance of DL-based models to classify the brain tumor.	This article may provide important insights to the researchers in the selection of the most suitable DL model following the specific application.	This study explored the DL methods used in the diagnosis of brain tumor patients. However, the meta-analysis does not explore the clinical implications of existing DL approaches for brain tumor classification.
The authors developed a new multiple-grading brain cell tissues classification strategy [50].	In this research, modified Deep-CNN architecture is employed with data augmentation to classify multi-grade brain tumors.	The suggested methodology may be utilized as an alternative to conventional multi-grade brain tumor segmentation.	This method is incapable to provide the desired accuracy in the case of the augmented datasets for brain cells tissues categorization.
In this paper, the authors acquired the multi-fold cross-checking of the brain cell tissues utilizing the novel deep learning algorithm [51].	In this research, a customized 3D-CNN is utilized to extract the features of brain tumors through MR imaging.	The suggested framework may be utilized to classify brain tumors at a growing rate than conventional methods.	The main limitation of the suggested work is lower contrasts in the segmentation phase of the MR images in real time.
In this article, the authors utilized another new deep learning algorithm rooted in the inception-V3 along with DensNet201 for brain cells tissues segmentation [52].	This suggested framework utilizes a customized DL algorithm and concatenation technique to attain improvised accuracy for brain tumor classification.	This suggested methodology may be employed as an alternative to conventional brain tumor diagnosis methods.	The key limitation of this suggested research is to remove entire features of the MR images from multifarious DensNet segments.
In the present work, the authors suggested a novel method of brain cells tissues segmentation rooted in the YOLO (You Only Look Once) strategy [53].	In this research, a customized DL algorithm is developed to extract features from brain tumor MR images.	This suggested method may determine brain tumors in the growing stage in some instances based on the size of the tumor than conventional methods.	The overall accuracy of this proposed method overall is only 85.95%, which is minimal. In addition to this, real-time implementation of the suggested model in modern systems is very intricate and consumes a large amount of time in the training phase which is another major drawback of this suggested approach.
In this work, the authors developed a new model based on the multiple-tasks generalization framework for brain cells tissues segmentation [54].	This suggested modified architecture is based on multi-task learning. The CNN classifier was trained for performing multiple tasks i.e., tumor grading as well as segmentation.	It is found that this suggested framework may be employed to segment multiple brain tumors than traditional approaches.	This method’s overall accuracy is low i.e., only 78% in the case of cells tissue segmentation of MR images.
In this work, the authors developed a novel framework for the cell’s tissue classification rooted in the RNN (Recurrent Neural Network) for the MR images [55].	This suggested framework utilizes modified RNNs to extract the features of tumors through MR imaging.	This suggested framework can be more reliable than conventional techniques for brain tumor categorization.	This proposed model’s accuracy is only 90% and the picture densities are low while performing the segmentations of the MR images.
In this work, authors utilized the combinations of multiple strategies such as the AlexNet and VGGNet based on the CNN for brain cells tissues segmentation [56].	In this research, a customized deep CNN architecture rooted in ResNet50 as well as transfer learning is developed for improving brain tumor classification accuracy.	The suggested approach may be utilized in the diagnosis of brain tumor patients.	The main problem of this method is high computation complexities in the case of feature extraction from a large dataset in real time.
In this work, the authors used a conventional neural network approach along with the VGG-16-rooted DL architecture for brain tumor identification [57].	The suggested method is developed based on modified VGG-16 and transfer learning for effective brain tumor categorization.	This developed model may determine the brain tumor in the growing stage more than conventional methods.	This proposed approach has numerous limitations, namely more time intensive, has lower accuracy, and has a large maintenance cost, which demands more attention.
In this study, the authors developed an intelligent model for brain tumor classification using MR images. This developed model is rooted in the CNN as well as LSTM hybrid approach for brain tumor detection [58].	In this research, a new model has been developed by customizing a combination of multifarious techniques.	This suggested methodology may be utilized to evaluate and categorize MR images.	However, the training and validation loss of this proposed model is large which is a significant drawback of the proposed model and requires more attention for developing a new model for providing more tumor detection accuracy.
In this research, the authors presented, a transfer-developed model rooted in the conventional CNN approach. This developed model was trained and validated using MR images [59].	The suggested method involves a multi-class categorization approach, that provides a more accurate outcome than a binary classification method.	This methodology is open-source and may be freely utilized or customized by the researchers.	This developed model also has a few limitations which include a slightly lower accuracy level on large pictures, more time-intensive as well implementations and maintenance cost is high.

**Table 3:** Types of GANs that form GAN ensembles in our research [49].

Name of GANs	Working Principle of the GANs
**PGGAN**	The PGGAN is an addition to the GAN training procedure, which enables the generator prototypical to train along with constancy that could originate massive higher quality pictures. The PGGAN initializes the training procedure by beginning along with the small picture and after that multiple layer blocks are assembled in increasing order in such a manner that generator prototypical outcome upsurges and upsurges the discriminator prototypical input proportions till the required picture proportion is acquired. The PGGAN scheme has the capability of higher quality synthetic pictures, which are extremely accurate.
**DCGAN**	The DCGAN utilizes convolution-invert as well as convolution stratums in discriminator along with a generator, correspondingly. In the DCGAN model discriminator comprises the stride convolutional stratum, batch regularization stratum as well as LeakyRelu as an activated role. The DCGAN utilizes multifarious guidelines such as eliminating the full attached unseen stratums for deep construction.
**Cycle GAN**	The CycleGAN is kind of a prototypical whose primary objective is to resolve picture-to-picture translation issues. One objective of the picture-to-picture translation issue is to acquire mapping among the input picture and output picture by utilizing the training dataset of the line-up picture sets. The CycleGAN is capable to learn the mapping instead of a need for coupled input/output pictures and uses cycle-dependable adversarial networks.
**Info GAN**	The info-GAN is a category of the generative adversarial network. The info-GAN alters the GAN aim for encouraging it for learning interpretable as well as expressive illustrations. It is completed through the maximization of communal datasets in between stationary minor subcategories of GAN in mutable as well as monitoring.
**Conditional GAN**	The Conditional GAN may be defined as a deep learning-based technique in which certain prerequisite constraints are put in a particular position. In the conditional GAN approach, an extra constraint, namely ‘z’ is assembled into a generator to produce the resultant dataset. The multiple labels are similarly placed in discriminator input for assisting the discriminator in distinguishing real-time datasets from falsely produced datasets.

**Table 4:** Partition of Brain tumor Dataset into training, validation, and test samples [60].

Kind of Tumor	Types of Planes	Number of Images	Total Images	Training Data (60%)	Validation Data (20%)	Testing Data (20%)
Glioma	Sagittal	495	1426	855	285	286
	Coronal	437				
	Axial	494				
Meningioma	Sagittal	231	708	424	142	142
	Coronal	268				
	Axial	209				
Pituitary	Sagittal	320	930	558	186	186
	Coronal	319				
	Axial	291				

**Table 5:** Abbreviation used in the pre-processing algorithm for the Skull stripping.

Abbreviations	Definitions
I_MR_	Input brain images
I_MR bin_	Binary image
Conv	Biggest connected component
Conv’ _Kt_	Structuring element
I_MRIstripped_	Skull stripped image

**Table 6:** Abbreviation used in the pre-processing algorithm for the MRI picture contrast improvement.

Abbreviations	Definitions
I_MRIstripped_	Input picture
$I_{{MRI}^{c}}$	Binary image
$C_{t}$	Modified dilation
$D_{t}$	Set of displacement
$L_{t}$	Structuring component
${SE}_{t2}$	Contour smoothness
$I_{MRIdiff}$	Closing operation
$I_{MRIimproved}$	Contrast-improved picture

**Table 7:** Abbreviation used in Classification using Modulated-CNN.

Abbreviations	Definitions
Nk	Main dataset
Pt1	Trained image
Timage	Labeled as an input
Pt1 _right_	Image right side
Pt1 _left_	Image left side

**Table 8:** Dataset portioning ratio (Training: Validation: Test samples) for different performance metrics.

Performance Metrics	Dataset Partitioning Ratio in % (Training: Validation: Test)			
	60:20:20	50:30:20	50:40:10	40:30:30
Accuracy	98.8%	97.33%	96.77%	95.33%
Precision	98.45%	97.22%	95.92%	96.53%
Recall	97.2%	96.88%	95.18%	94.28%
F1-Score	98.11%	96.10%	95.44%	94.36%
NPV	98.09%	96.90%	94.94%	94.10%

**Table 9:** Performance Metric Evaluation of the proposed model on different diseases.

Disease Classification	Performance Metrics				
	Accuracy	Precision	Recall	F1-Score	NPV
Skin Cancer	96.45%	96.11%	94.33%	95.85%	95.73%
Lung Cancer	95.88%	96.45%	95.79%	96.74%	94.78%
Breast Cancer	96.73%	95.71%	95.12%	97.04%	96.99%
Prostate cancer	97.25%	97.33%	96.71%	95.88%	97.02%
Brain Tumor	98.8%	98.45%	97.2%	98.11%	98.09%

**Table 10:** Cumulative analysis of performance metrics for different types of GANs.

Types of GANs	Performance Metrics				
	Accuracy	Precision	Recall	F1-Score	NPV
PGGAN	98.8%	98.45%	97.2%	98.11%	98.09%
DCGAN	97.24%	96.14%	95.15%	96.01%	96.55%
Cycle-GAN	96.18%	95.12%	95.88%	96.03%	95.77%
Info-GAN	92.15%	91.22%	91.99%	91.77%	91.05%
Conditional-GAN	90.24%	88.02%	90.14%	89.33%	89.09%

## Data Availability

Authors utilized publicly available datasets [60].
